# Longitudinal ctDNA monitoring in patients with metastatic uveal melanoma undergoing isolated hepatic perfusion in combination with ipilimumab and nivolumab

**DOI:** 10.1016/j.iotech.2025.101079

**Published:** 2025-10-26

**Authors:** M. Kadefors, A. Nelson, E. Blomberg, A. Ståhlberg, L. Ny, R. Olofsson Bagge

**Affiliations:** 1 Department of Surgery, Institute of Clinical Sciences, Sahlgrenska Academy, University of Gothenburg, Gothenburg, Sweden; 2 Department of Oncology, Institute of Clinical Sciences, Sahlgrenska Academy, University of Gothenburg, Sahlgrenska University Hospital, Gothenburg, Sweden; 3 Oncobit AG, Schlieren, Switzerland; 4 Department of Laboratory Medicine, Institute of Biomedicine, Sahlgrenska Academy, University of Gothenburg, Gothenburg, Sweden; 5 Wallenberg Centre for Molecular and Translational Medicine, University of Gothenburg, Gothenburg, Sweden; 6 Region Västra Götaland, Sahlgrenska University Hospital, Department of Clinical Genetics and Genomics, Gothenburg, Sweden; 7 Science for Life Laboratory, Institute of Biomedicine, University of Gothenburg, Gothenburg, Sweden; 8 Department of Surgery, Sahlgrenska University Hospital, Gothenburg, Sweden

**Keywords:** circulating tumor DNA, isolated hepatic perfusion, immune checkpoint inhibitor, liver metastases, uveal melanoma

## Abstract

**Background:**

Uveal melanoma (UM) is a rare cancer, which often metastasizes to the liver, leading to a poor prognosis. In the randomized SCANDIUM II trial, patients with metastatic UM received a one-time treatment with isolated hepatic perfusion using high-dose melphalan in combination with systemic immune checkpoint inhibition with ipilimumab (3 mg/kg) and nivolumab (1 mg/kg). In this study, we assessed circulating tumor DNA (ctDNA) in prospectively collected blood samples from all patients in the SCANDIUM II trial to detect residual disease and recurrence as a strategy to evaluate treatment efficacy and predict prognosis.

**Patients and methods:**

We analyzed ctDNA in 128 plasma samples from 18 patients using digital PCR (Oncobit™ PM) targeting GNA11 Q209L (*n* = 9), GNAQ Q209P (*n* = 5), or GNAQ Q209L (*n* = 4).

**Results:**

At baseline, ctDNA was detectable in 9 of 17 (53%) patients, with levels positively correlated with lactate dehydrogenase and tumor burden. Analysis of ctDNA dynamics revealed ctDNA increase before progression in a subset of patients (5 of 12 patients, 42%). After treatment initiation, six of seven (86%) patients showed ctDNA clearance or decrease. Patients with undetectable ctDNA 2-4 months after the start of treatment had significantly improved progression-free survival (*P* = 0.024), and a non-significant improvement in overall survival.

**Conclusions:**

In patients with UM liver metastases treated with combined hepatic perfusion and immune checkpoint inhibition, ctDNA may serve as a predictive biomarker and warrants further validation.

## Highlights

• Baseline ctDNA levels in patients with metastatic UM correlated with tumor burden.
• In patients receiving hepatic perfusion and immunotherapy, ctDNA dynamics reflected disease activity and may guide care.
• Early on-treatment ctDNA status was associated with PFS.

## Introduction

Uveal melanoma (UM) is a rare form of melanoma that arises in the eye. Approximately 50% of patients eventually develop metastatic disease.^1^ In this setting, the organ distribution of metastases is strongly hepatotropic, with isolated liver metastases observed in over half of patients. The prognosis is poor, with a median overall survival (OS) of ∼1 year after diagnosis of metastatic disease.^1^^,^^2^

Immune checkpoint inhibitors (ICIs) have shown limited clinical efficacy in metastatic UM. Combination therapy with ipilimumab and nivolumab yields overall response rates (ORRs) of 10%-18%, with an uncertain impact on OS.^3^^,^^4^ Tebentafusp, a bispecific fusion protein that redirects T cells to target melanoma cells, demonstrated a significant OS benefit (22 versus 16 months) in a randomized phase III trial in HLA-A∗02:01-positive patients, despite modest response rates as assessed by radiology.^5^

Isolated hepatic perfusion (IHP) with melphalan is a regional therapy that involves surgical isolation of the liver from the systemic circulation, enabling delivery of high-dose chemotherapy directly to the liver with minimal systemic exposure. The effective melphalan dose delivered to the liver during IHP is ∼10 times higher than the dose used in myeloablative conditioning regimens for hematological malignancies. In the randomized phase III SCANDIUM trial, we reported a significantly higher ORR (40% versus 4%), and a numerically longer median OS (21.7 versus 17.6 months) with IHP compared with best alternative care in treatment-naive patients with isolated liver metastases from UM.^6^^,^^7^

In the phase Ib SCANDIUM II trial (NCT04463368), we combined IHP with ICIs,^8^ where 18 patients with liver metastases from UM received a single IHP treatment combined with up to four cycles of ipilimumab (3 mg/kg) and nivolumab (1 mg/kg). Among treated patients, the ORR was 43%. The study also evaluated a pre/post-operative strategy where patients were randomly assigned to receive either one cycle of IPI3/NIVO1 before IHP (*n* = 9), followed by three additional cycles post-operatively, or first IHP and then four cycles of IPI3/NIVO1 (*n* = 9). The hypothesis was that pre-treatment with ICIs would enhance efficacy, albeit with increased risk of toxicity. The results, however, showed that the post-operative-only strategy was not only better tolerated but also associated with a numerically higher ORR (57% versus 22%) and longer progression-free survival (PFS, 11.8 versus 6.0 months).

The gold standard for assessing treatment-induced tumor response is CT imaging evaluated using RECIST criteria.^9^ This method may underestimate therapeutic benefit, however, particularly in the context of ICI treatment, where pseudoprogression can occur. To address this limitation, immune-related RECIST (irRECIST) was developed to better capture atypical response patterns.^10^^,^^11^ Circulating tumor DNA (ctDNA) has emerged as a promising blood-based biomarker for detecting minimal residual disease and monitoring therapy response, with levels shown to correlate with both tumor burden and disease progression.^12^^,^^13^ As was seen in the phase III trial of tebentafusp, radiological responses were uncommon despite clear survival benefits, suggesting that conventional imaging may not completely reflect therapeutic efficacy. Instead, ctDNA showed potential as a superior prognostic marker in the same trial.^14^

Digital PCR (dPCR) enables highly sensitive detection of ctDNA using mutation-specific assays. In UM, mutually exclusive mutations in GNAQ and GNA11, predominantly at codon 209 (Q209L and Q209P), are the most common genetic alterations.^15^^,^^16^ These tumor-specific mutations represent stable molecular targets suitable for ctDNA analysis. In cutaneous melanoma, early changes in ctDNA levels have been associated with response to ICIs, and several studies have suggested that a rapid decline or clearance of ctDNA during treatment may serve as an early predictor of therapeutic benefit and improved survival outcomes.^17^ These findings support the use of ctDNA as a dynamic biomarker for monitoring ICI response. The predictive or prognostic value of ctDNA in the context of regional high-dose chemotherapy, such as IHP, however, remains unknown. It is also unclear how combination strategies involving IHP and ICIs influence ctDNA kinetics.

In this study, we analyzed ctDNA levels in patients treated within the SCANDIUM II trial to evaluate the potential of ctDNA as a clinical tool to guide treatment with IHP in combination with ICI.

## Patients and methods

### Study design/patients

The SCANDIUM II trial^8^ (NCT04463368) was a Swedish prospective, multicenter, open-label, phase Ib trial randomizing patients in a 1 : 1 ratio to receive either IHP followed by combination immunotherapy with four cycles of ipilimumab 3 mg/kg and nivolumab 1 mg/kg (IPI3/NIVO1) every 3 weeks (post-operative arm), or one pre-operative cycle of IPI3/NIVO1 before IHP followed by three cycles of IPI3/NIVO1 (pre/post-operative arm). Both arms thereafter received monotherapy with nivolumab 480 mg every 4 weeks for up to 1 year. IHP was carried out at Sahlgrenska University Hospital, Gothenburg, Sweden. The liver was perfused with melphalan at a dose of 1 mg/kg body weight for 1 h, as previously described.^18^ The baseline characteristics of the patients are shown in Table 1. The study was approved by the Swedish Ethical Review Authority (Dnr 2021-05391-02), and conducted in accordance with the protocol, Good Clinical Practice guidelines and the provisions of the Declaration of Helsinki. All patients provided written informed consent before inclusion in the trial.

**Table 1:** Baseline patient characteristics

	Post-op (*n* = 9)	Pre/post-op (*n* = 9)
Age, median (range), years	62 (48-73)	65 (54-70)
Sex, *n* (%)		
Female	1 (11)	5 (56)
Male	8 (89)	4 (44)
ECOG PS, *n* (%)		
0	9 (100)	8 (89)
1	0 (0)	1 (11)
Previous treatment of metastatic disease, *n* (%)		
No previous treatment	9 (100)	5 (56)
Chemotherapy^a^	0	3 (33)
Surgical resection	0	1 (11)
Radiotherapy	0	1 (11)
Largest metastatic lesion, *n* (%)		
≤3.0 cm (M1a)	6 (67)	7 (78)
3.1-8.0 cm (M1b)	3 (33)	0 (0)
≥8.1 cm (M1c)	0 (0)	2 (22)
Mean tumor volume, % (range %)	11 (1-45)	11 (5-40)
Metastatic sites, *n* (%)		
Isolated liver metastases	8 (89)	5 (56)
Liver and extrahepatic metastases	1 (11)	4 (44)
Years since primary diagnosis, median (range)	0.9 (0.2-6.8)	2.2 (0.1-10.9)
Lactate dehydrogenase > ULN, *n* (%)	5 (56)	5 (56)

ECOG PS, Eastern Cooperative Oncology Group performance status; IQR, interquartile range; op, operative; ULN, upper limit of normal.

a Temozolomide.

### Sample collection and cell-free DNA isolation

Blood samples were prospectively collected in cell-free DNA BCT tubes (Streck, 218997) and cell-free plasma was extracted using a two-step centrifugation protocol (300 × *g* for 20 min and 5000 × *g* for 10 min), after which samples were stored at −80°C. Cell-free DNA was isolated from 2 ml of plasma using the QIAamp Circulating Nucleic Acid Kit (Qiagen) together with the QIAvac 24 Plus vacuum system, according to the manufacturer’s protocol. The concentration of eluted cell-free DNA was measured using the Qubit 4.0 Fluorometer (Life Technologies).

### Detection of recurrent GNAQ/GNA11 mutations by digital PCR

Plasma cell-free DNA samples were analyzed using dPCR (Oncobit™ PM, Oncobit AG, Schlieren, Switzerland), targeting the hotspot mutations GNA11 Q209L, GNAQ Q209L, or GNAQ Q209P. Determining which Oncobit™ PM test used for each patient was based on whole-genome sequencing analysis of patient tumors. For two patients without whole-genome sequencing data, all three tests were run sequentially until one of the tests showed detectable ctDNA. For droplet dPCR, up to 36 ng of cell-free DNA was added per reaction, combined with ddPCR Supermix for Probes (No dUTP) (Bio-Rad, Hercules, CA) and the primer–probe mix of one of the Oncobit™ PM assays (Oncobit AG). Each run included a no-template control and the appropriate Oncobit™ PM Control as a positive control. Droplet generation and PCR amplification were carried out on the Bio-Rad QX200 AutoDG Droplet Digital PCR System, following manufacturer and assay-specific protocols.

Fluorescence signals were acquired using the QX Manager Software Standard Edition (version ≥2.0, Bio-Rad), and data were processed with the Oncobit™ PM Analyzer software (version 1.1), which applies an empirically optimized algorithm for ctDNA detection, sample classification and quantification. Mutant allele frequency (MAF, expressed as a percentage) as determined by the Oncobit™ PM Analyzer was used for all reported analyses.

Clearance of ctDNA was defined as a transition from detectable to undetectable ctDNA levels. ctDNA decrease was defined as a significant reduction in MAF, with non-overlapping 95% confidence intervals (CIs) reflecting single-sample assay uncertainty. A ctDNA increase was defined as a significant rise in MAF, with non-overlapping 95% CIs, or as the detection of ctDNA following a previously undetectable sample. An increase in ctDNA was further classified as a confirmed increase when followed by a non-decreasing positive sample, and as unconfirmed increase otherwise.

### Statistical analysis

Statistical analysis was carried out using R Statistical Software (version 4.5.0, R Core Team 2025). Correlation between ctDNA and LDH levels was evaluated using Pearson correlation coefficient. Data was described by median, and the Wilcoxon rank-sum test was used to assess differences. Kaplan–Meier plots with log-rank tests were used to assess the differences in survival distributions between stratified patient groups.

## Results

**Figure 1:**
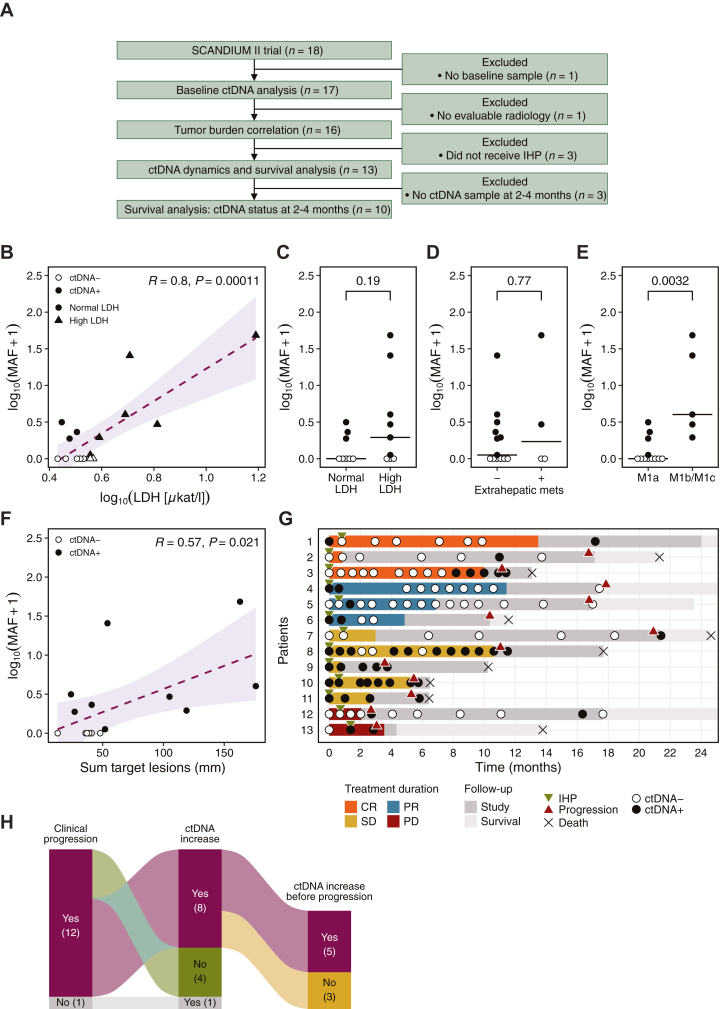
**Baseline ctDNA analysis and longitudinal tracking.** (A) Diagram showing patient exclusion criteria for different ctDNA analyses. (B-E) Associations between baseline ctDNA levels and clinical variables. (B) Correlation between ctDNA MAF (%) and LDH levels at baseline (*n* = 17). Correlations were assessed using Pearson correlation. (C) Baseline ctDNA MAF in patients with normal versus high LDH levels (high defined as LDH > ULN, *n* = 17). (D) Baseline ctDNA MAF in patients without versus with extrahepatic metastases (*n* = 17). (E) Baseline ctDNA MAF in patients with stage M1a versus M1b or M1c disease (*n* = 17). Lines represent median. The Wilcoxon rank-sum test was used to compare groups. (F) Correlation between ctDNA MAF (%) and tumor burden according to RECIST (sum target lesions) baseline (*n* = 16). Correlations were assessed using Pearson correlation. (G) Swimmer plot depicting clinical events and ctDNA status for patients who received IHP (*n* = 13) at various timepoints over the course of treatment and follow-up. Treatment duration lines are colored according to best overall response. (H) Sankey diagram illustrating the relationship between clinical progression, ctDNA increase after treatment start and timing of ctDNA increase (before or at the time of clinical progression) in the 13 patients with evaluated ctDNA dynamics. CR, complete response; IHP, isolated hepatic perfusion; LDH, lactate dehydrogenase; MAF, mutant allele frequency; PD, progressive disease; PR, partial response; SD, stable disease.

In total, 128 ctDNA samples from 18 patients with metastatic UM undergoing combined treatment in the SCANDIUM II trial were analyzed retrospectively by dPCR for the presence of the hotspot mutations GNA11 Q209L (*n* = 9, 50%), GNAQ Q209P (*n* = 5, 28%), and GNAQ Q209L (*n* = 4, 22%). One patient without a baseline sample was excluded from all analysis, and one patient without evaluable radiology was not assessed for tumor burden as defined by RECIST (Figure 1A). The 13 patients who received both IHP and ICI treatment and had both evaluable radiology and a baseline sample were included for ctDNA dynamics and outcome analysis (Figure 1A).

### Baseline ctDNA and clinical characteristics

Nine out of 17 (53%) patients were ctDNA-positive at baseline. Baseline ctDNA MAF positively correlated with lactate dehydrogenase (LDH) levels (Figure 1B), but ctDNA levels did not show any statistical increase when comparing patients with elevated (LDH > ULN), and normal LDH levels (Figure 1C). The presence of extrahepatic metastases was not associated with higher ctDNA levels (Figure 1D). Patients with higher metastatic burden (M1b and M1c), according to tumor–node–metastasis staging, had higher ctDNA levels at baseline than patients with lower metastatic burden (M1a), and all patients with M1b or M1c had detectable ctDNA (Figure 1E). In line with this, ctDNA levels were positively correlated with RECIST-defined tumor burden, as assessed by the sum of the diameter of target lesions (Figure 1F).

### ctDNA dynamics and treatment response

**Figure 2:**
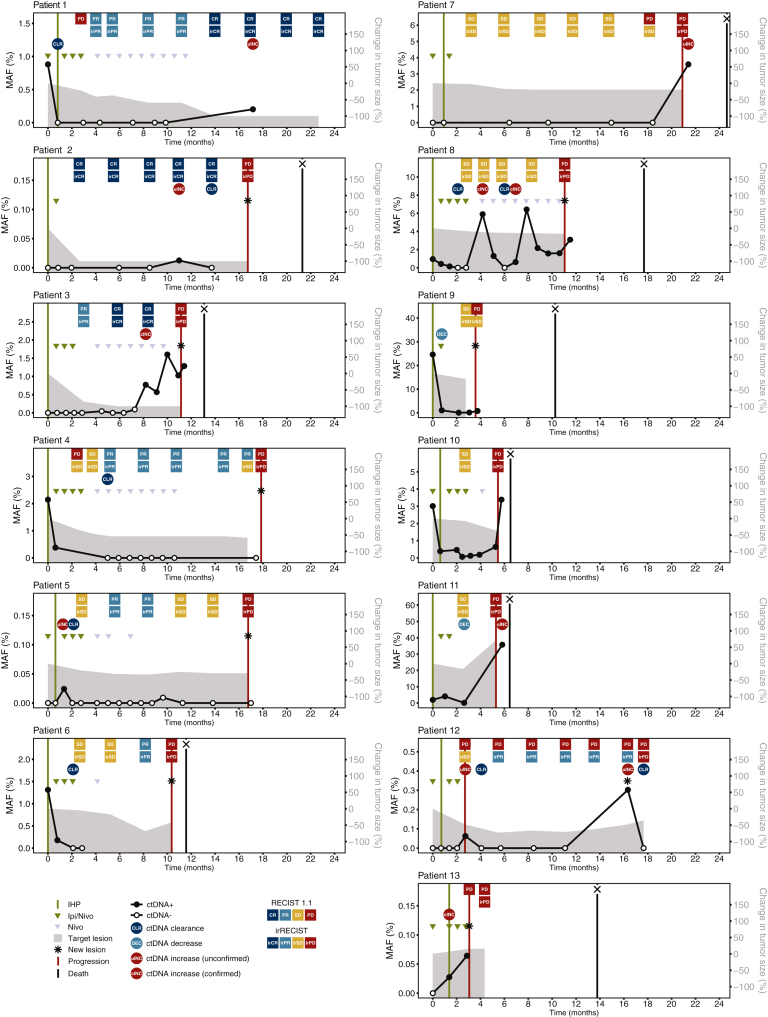
**Patient-specific longitudinal ctDNA dynamics and tumor response.** Plots are shown for patients who received IHP and had evaluable radiology (*n* = 13). Plots show MAF (%) on the left *y*-axis (black line) and change in target lesion size (% change from baseline) on the right *y*-axis (gray area under the curve). CR, complete response; IHP, isolated hepatic perfusion; Ipi, ipilimumab; irCR, immune-related complete response; irPD, immune-related progressive disease; irPR, immune-related partial response; irSD, immune-related stable disease; MAF, mutant allele frequency; Nivo, nivolumab; PD, progressive disease; PR, partial response; SD, stable disease.

Individual ctDNA dynamics together with radiological assessment and clinical events are shown in Figure 2. A median of 8 longitudinal samples (range 3-15) was analyzed for each of these 13 patients with the last sample taken at a median of 11.4 months (range 0.7-21.4 months) after treatment initiation (Figure 1G). Six out of seven (86%) patients who were ctDNA-positive at baseline showed ctDNA clearance (*n* = 4, 57%) or decrease (*n* = 2, 29%) at some time point after treatment initiation. Three out of four (75%) patients who cleared ctDNA had a best overall response (BOR) of complete response (CR, *n* = 1) or partial response (PR, *n* = 2), while the fourth patient had stable disease (SD). Additionally, the two patients with decreased ctDNA and the patient with stable ctDNA all had SD as BOR. At a fixed early timepoint (3 months), the distribution of ctDNA responses was broadly consistent with BOR, as illustrated in Supplementary Figure S1A, available at https://doi.org/10.1016/j.iotech.2025.101079.

Twelve out of thirteen patients (92%) showed clinical progression before the end of follow-up (Figure 1G). Out of these 12 patients, 8 (67%) patients also showed ctDNA increase at some time point after the start of treatment (Figure 1H). One patient who completed follow-up without clinical progression showed ctDNA increase in the last sample taken at 17.1 months (5 months before end of study, Figure 2). An increase in ctDNA levels was observed before clinical progression for 5 out of 12 (42%) patients, and three of these patients had confirmed ctDNA increase as determined by a subsequent non-decreasing positive ctDNA sample (Figures 1H and 2). One patient (Pt 5) showed early ctDNA increase (41 days after treatment start) followed by clearance, which is likely unrelated to the disease progression observed 15 months later (15.4 months after ctDNA increase). Out of the four patients who showed no increase in ctDNA during the follow-up period, two patients showed ctDNA clearance after start of treatment and two patients remained ctDNA-positive after treatment start. Of note, one patient (Pt 6) who did not show a ctDNA increase had missing follow-up samples in the 7 months before progression.

Three patients had progressive disease according to RECIST 1.1 criteria at the first evaluation. Treatment with ICI was continued beyond progression, as permitted by the study protocol, until a confirmed progression was observed. These three patients ultimately achieved a BOR of irCR (*n* = 1), or irPR (*n* = 2), according to irRECIST criteria. ctDNA dynamics at the time of initial radiological progression were discordant: one patient (Pt 1) showed a decrease in ctDNA, one (Pt 12) had a transient increase, and one (Pt 4) had no timely available ctDNA (Figure 2).

### Undetectable ctDNA at early follow-up is associated with progression and survival

**Figure 3:**
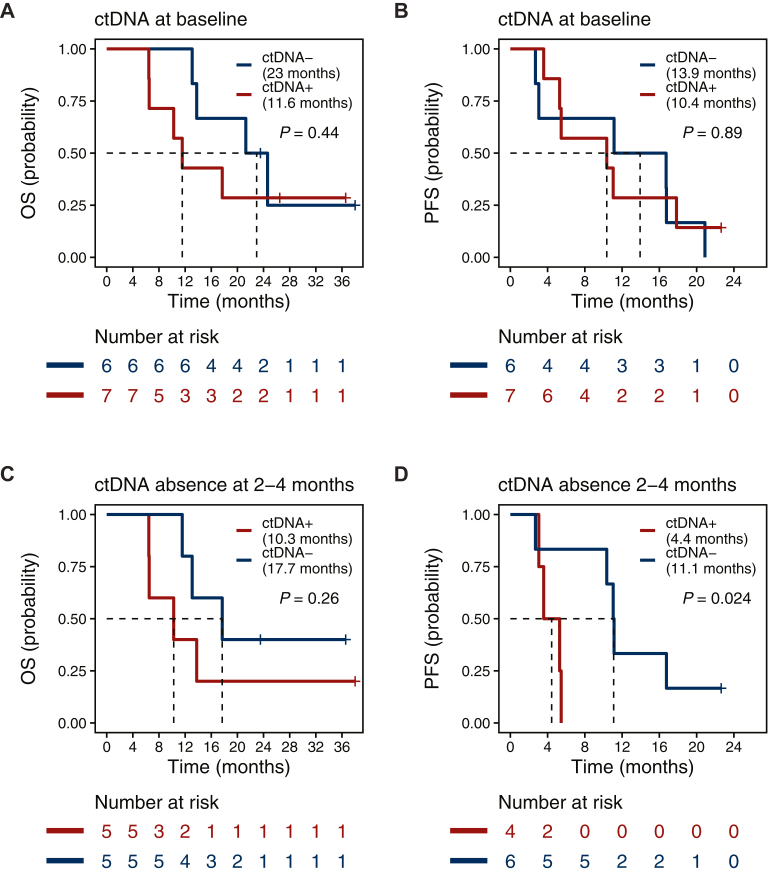
**Survival analysis (OS and PFS) of patients who received IHP stratified by ctDNA status.** (A) Overall survival of patients with ctDNA− baseline sample (*n* = 6) compared with ctDNA+ baseline sample (*n* = 7). (B) Progression-free survival of patients with ctDNA− baseline sample (*n* = 6) compared with ctDNA+ baseline sample (*n* = 7). (C) Overall survival of patients without detected ctDNA (ctDNA−) 2-4 months after treatment start (*n* = 5) compared with patients with at least one positive ctDNA sample (ctDNA+) 2-4 months after treatment start (*n* = 5). (D) Progression-free survival of patients without detected ctDNA (ctDNA−) 2-4 months after treatment start (*n* = 6) compared with patients with at least one positive ctDNA sample (ctDNA+) 2-4 months after treatment start (*n* = 4). Only samples taken before progression within the 2-4-month time frame were evaluated. Kaplan–Meier plots were used for survival analysis and survival distributions were compared using log-rank test. OS, overall survival; PFS, progression-free survival.

For patients who received IHP, there was a numerical predictive value of ctDNA status at baseline on OS, with a median OS of 23 months for patients with undetectable ctDNA and 11.6 months for patients with detectable ctDNA, though this difference was not statistically significant (Figure 3A). No effect on PFS was observed (Figure 3B). Radiological assessment of treatment response at 3 months is common. We hypothesized that ctDNA status between 2 and 4 months after treatment initiation could serve as a prognostic biomarker. Patients who were ctDNA-negative during this window showed a non-significant trend toward improved OS (median OS: 17.7 months for ctDNA-negative versus 10.3 months for ctDNA-positive, Figure 3C). ctDNA negativity was significantly associated with improved PFS (*P* = 0.024), with median PFS of 11.1 months for ctDNA-negative patients compared with 4.4 months for those who were ctDNA-positive (Figure 3D). Furthermore, out of the patients with samples taken between 2 and 4 months after treatment start, all responders (CR, n = 2; PR, n = 2) had undetectable ctDNA and five out of six (83%) non-responders (SD, n = 4; PD, n = 2) had detectable ctDNA at 2-4 months (Figure 1G). Although based on a very small number of patients with evaluable ctDNA response (*n* = 6), ctDNA clearance at 3 months showed a numerical trend toward improved OS and PFS (Supplementary Figure S1B and C, available at https://doi.org/10.1016/j.iotech.2025.101079).

## Discussion

This is the first study to explore ctDNA as a biomarker for disease monitoring and prognosis in patients with metastatic UM undergoing combined treatment with IHP and ICI. Our findings demonstrate that ctDNA levels correlate with tumor burden, and that ctDNA status at early follow-up (2-4 months) is associated with disease progression, highlighting the potential of ctDNA as a useful biomarker to support clinical decision making in this patient population.

At baseline, ctDNA was detectable in 53% of assessable patients, a detection rate slightly lower but comparable with the 61% reported in two previous studies.^13^^,^^14^ Baseline ctDNA levels were significantly associated with tumor burden as reflected by the RECIST-defined sum of target lesions, and with established markers including radiological M-stage and LDH levels. Notably, all patients with M1b or M1c disease were ctDNA-positive, whereas a subset of patients with lower metastatic burden (M1a) had non-detectable ctDNA. This supports the hypothesis that ctDNA detection reflects tumor burden and may be more likely elevated in patients with extensive disease activity. It also highlights the need for more sensitive detection methods in patients with lower tumor burden.

Previous studies in metastatic UM have demonstrated that patients with lower or undetectable baseline ctDNA have better survival outcomes.^13^^,^^14^^,^^19^^,^^20^ In line with these studies, we observed a numerical improvement in OS for patients with undetected ctDNA, but the effect was not significant. This trend is consistent with previous findings but the small sample size of this phase Ib trial limits our ability to demonstrate significant relationships.

Following initiation of combined IHP and ICI, ctDNA clearance was observed in 57% of patients who were ctDNA-positive at baseline, and 75% of these patients demonstrated an objective response to treatment. A similar pattern between ctDNA clearance and treatment response was also observed at the fixed 3-month timepoint. Importantly, undetectable ctDNA at 2-4 months post-treatment initiation was significantly associated with improved PFS and trended toward improved OS. This observation is consistent with prior studies in UM linking on-treatment ctDNA clearance or reduction to improved survival,^13^^,^^14^ including a pivotal tebentafusp trial, where ctDNA decrease was shown to outperform radiological assessments in predicting long-term outcomes.^20^ Together, these data suggest that early ctDNA clearance may predict durable treatment response, and undetectable ctDNA 2-4 months post-treatment could serve as an early surrogate. Conversely, persistent detection or increasing ctDNA at this timepoint could signal the need for closer surveillance or second-line treatment strategies.

Longitudinal ctDNA monitoring also enabled detection of disease progression earlier than imaging in a subset of patients. In total, 42% of patients with clinical progression exhibited rising ctDNA levels before radiographical evidence of disease progression. This illustrates the potential utility of ctDNA for earlier identification of treatment failure and timely clinical intervention. Increases in ctDNA, however, were not observed before clinical progression in the remaining patients, thus suggesting that while ctDNA monitoring can provide early molecular insight, its clinical utility for detecting progression may be influenced by both biological and practical factors, such as sampling frequency and assay sensitivity, and requires further validation.

Despite these promising findings, several challenges remain. As mentioned previously, not all patients with disease progression exhibited a preceding ctDNA increase, and some showed discordant ctDNA and imaging dynamics, such as late ctDNA rise without progression or transient ctDNA spikes. In the phase II PEMDAC trial of pembrolizumab and entinostat in metastatic UM, similar complex longitudinal patterns were observed, with ctDNA spikes speculated to reflect waves of tumor proliferation.^19^ A subset of patients remained ctDNA-negative at all timepoints, despite having radiologically detectable disease. These inconsistencies may reflect biological heterogeneity, variability in ctDNA shedding, anatomical barriers to ctDNA release, or technical limitations in ctDNA detection.^21^^,^^22^ Although dPCR is highly sensitive, it may miss ctDNA in samples with ultra-low levels or in cases involving non-targeted subclonal variants, although the latter is not expected to occur when retained truncal mutations are targeted, such as GNAQ/GNA11 in UM. Broader panels may improve detection and reveal resistance mechanisms and tumor evolution by increasing the number of molecular targets and capturing additional subclonal variants. Additionally, longitudinal sampling was carried out with a median of eight timepoints per patient. Some samples were missing, particularly during the post-treatment follow-up period. Variability in sample collection timing further complicated alignment with imaging data. This, together with the limited plasma input volume (2 ml per sample) available for analysis, may have limited detection of key ctDNA changes, such as nadir or resurgence.

This study also has important limitations which affect the generalizability of our findings. Most notably, the small sample size (*n* = 13 assessable patients) limits the statistical power and precludes definitive conclusions. This is a well-known challenge in rare cancers such as UM, but nevertheless emphasizes the need for larger, multicenter validation cohorts.

Despite these limitations, our findings support the clinical utility of ctDNA monitoring in patients with UM undergoing liver-directed and systemic immunotherapy. Future prospective studies are warranted to validate these findings, define standardized thresholds for interpretation, and explore integration of ctDNA with other biomarkers such as LDH, imaging features, and immune signatures, for a more comprehensive assessment of treatment response.

In conclusion, this exploratory biomarker analysis in the SCANDIUM II trial supports the role of ctDNA as a promising biomarker for real-time monitoring of therapeutic response and outcome prediction in metastatic UM. Undetectable ctDNA at early follow-up following intervention is associated with improved survival and may support clinical decision making beyond conventional imaging. As ctDNA technologies evolve, their integration into the management of UM could enable more personalized care strategies for this challenging disease.
