# Supplementary material for: Longitudinal ctDNA monitoring in patients with metastatic uveal melanoma undergoing isolated hepatic perfusion in combination with ipilimumab and nivolumab
Source: Immunooncol Technol. 2025 Oct 26;28:101079. doi: 10.1016/j.iotech.2025.101079 (PMC12682135; doi:10.1016/j.iotech.2025.101079)
Supplement: Supplementary Data [file mmc1.pdf]

Supplementary Data for

Longitudinal ctDNA monitoring in patients with metastatic uveal melanoma undergoing isolated hepatic perfusion in combination with ipilimumab and nivolumab

Måns Kadefors, Axel Nelson, Evelina Blomberg, Anders Ståhlberg, Lars Ny,

Roger Olofsson Bagge

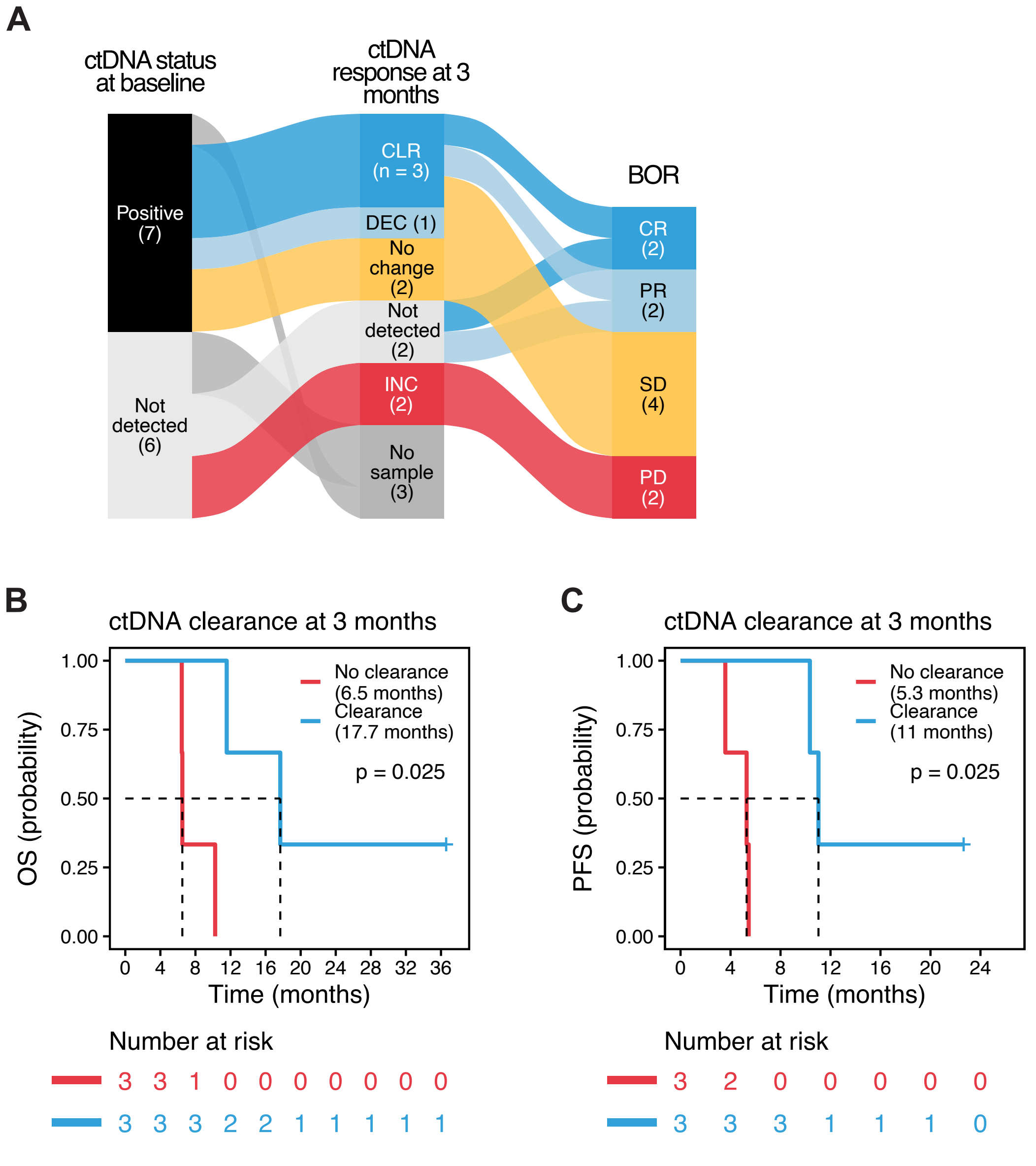

Supplementary Figure 1. Radiological response and survival of patients stratified by ctDNA response at 3 months. (**A**) Sankey diagram illustrating the relationship between baseline ctDNA status, ctDNA response at 3 months, and RECIST-based best overall response. Among the 13 patients who received isolated hepatic perfusion and had baseline ctDNA and evaluable radiology, 6 patients with detectable baseline ctDNA and a 3-month sample were evaluable for ctDNA clearance/reduction, 2 showed ctDNA emergence at 3 months, and 5 were not evaluable due to missing 3-month samples or undetected ctDNA at both timepoints. (**B**) Overall survival and (**C**) progression-free survival of patients with ctDNA clearance (n = 3) versus no clearance (n = 3) at 3 months compared to baseline. CLR: ctDNA clearance; DEC: ctDNA decrease; INC: ctDNA increase/emergence; BOR: best overall response; CR: complete response; PR: partial response; SD: stable disease; PD: progressive disease, OS: overall survival; PFS: progression-free survival.
